# Population pharmacokinetics and dose optimization of magnesium sulfate in Chinese preeclampsia population

**DOI:** 10.1186/s12884-024-06620-x

**Published:** 2024-06-13

**Authors:** Jing Deng, Lan Peng, Yuwei Wang, Jingjing Li, Lian Tang, Yanxia Yu

**Affiliations:** 1 The Affiliated Suzhou Hospital of Nanjing Medical University, Gusu School, Suzhou, Jiangsu 215002 China; 2 Department of Pharmacy, The Affiliated Suzhou Hospital of Nanjing Medical University, Suzhou Municipal Hospital, Suzhou, Jiangsu 215002 China; 3 Department of Obstetrics, The Affiliated Suzhou Hospital of Nanjing Medical University, Suzhou Municipal Hospital, Suzhou, Jiangsu 215002 China

**Keywords:** Magnesium sulfate, Preeclampsia, Population pharmacokinetics, Monte Carlo simulation, Dose optimization

## Abstract

**Objective:**

To establish the population pharmacokinetics (PPK) of magnesium sulfate (MgSO_4_)in women with preeclampsia (PE), and to determine the key covariates having an effect in magnesium pharmacokinetics in Chinese PE.

**Methods:**

Pregnant women with PE prescribed MgSO4 were enrolled in this prospective study from April 2021 to April 2023. On the initial day of administration, the patients were administered a loading dose of 5 g in conjunction with 10 g of magnesium sulfate as a maintenance dose. On the second day, only the maintenance dose was administration, and maternal blood samples were taken at 0, 4, 5, and 12 h after the second day’s 10 g maintenance dose. The software Phoenix was used to estimate PPK parameters of MgSO4, such as clearance (CL) and volume of distribution (V), and to model PPK models with patient demographic, clinical, and laboratory covariates.

**Results:**

A total of 199 blood samples were collected from 51 women with PE and PPK profiles were analyzed. The PPK of MgSO_4_ is consistent with to a one-compartment model. The base model adequately described the maternal serum magnesium concentrations after magnesium administration. The population parameter estimates were as follows: CL was 2.98 L/h, V was 25.07 L. The model predictions changed significantly with covariates (BMI, creatinine clearance, and furosemide). Furosemide statistically influences V. The creatinine clearance, BMI and furosemide jointly affects CL. Monte Carlo simulation results showed that a loading dose combined with a maintenance dose would need to be administered daily to achieve the therapeutic blood magnesium concentrations. For the non-furosemide group, the optimal dosing regimen was a 5 g loading dose combined with a 10 g maintenance dose of MgSO4. For the furosemide group, the optimal dosing regimen was a 2.5 g loading dose combined with a 10 g maintenance dose of MgSO4.

**Conclusions:**

The magnesium PPK model was successfully developed and evaluated in Chinese preeclampsia population, and the dose optimization of MgSO_4_ was completed through Monte Carlo simulation.

## Background

PE is a progressive disease of pregnancy involving multiple organ systems affect 2–8% of all pregnancies globally [1, 2]. They are the primary causes of the rise in preterm labor, maternal morbidity, and perinatal mortality worldwide. Currently, there is no cure for this pregnancy disorder and supportive care can only be achieved by using antihypertensive drugs and MgSO_4_ to control blood pressure and prevent the occurrence of eclampsia, which slows disease progression and hopefully prolongs the pregnancy [3]. MgSO_4_ is considered safe and cost-effective by the World Health Organization and is currently the anticonvulsant of choice for the prevention and control of eclamptic fits [4].

In spite of the availability of a clinical protocol for treatment of PE and eclampsia such as Zuspan (IV) and Pritchard (IV and IM) regimen, the international actual regimens in use varied widely [5]. Loading doses of 4–6 g intravenously over 20–30 min are typically used, followed by maintenance doses of 1–2 g/h (and up to 3 g/h) [6]. Although therapeutic serum concentrations of magnesium for the prevention and treatment of eclampsia have not been rigorously determined, MgSO_4_ has a narrow therapeutic margin. Too low a dose increases the risk of convulsions, while too high a dose can result in maternal/fetal toxicity. However, there are multiple literature reports indicating that multiple MgSO_4_ administration schemes cannot achieve the level of concentration that is considered to have therapeutic effects (2.0–3.5 mmol/L) [7]. In 2018, our team conducted a retrospective survey on patients with PE who were treated with MgSO_4_. The survey found that only 31.5% of patients reached the effective treatment concentration range within 24 h of administration [8]. The design of regimen to achieve and maintain the desired serum magnesium concentration necessitates essential pharmacokinetic parameters, including CL and V. However, the available pharmacokinetic data on magnesium are severely limited, with significant variability observed across different countries. Therefore, the aim of this study is to develop an accurate PPK model based on the Chinese dosing regimen to assess the impact of potentially influential covariates on the magnesium pharmacokinetics and to individualize according to the basic characteristics to maximize the therapeutic benefit while minimizing toxicity.

## Methods

### Patients and data collection

This was an observational, longitudinal, prospective study to evaluate the PPK of Magnesium in PE, conducted between April 2021 and April 2023 at the Affiliated Suzhou Hospital of Nanjing Medical University in China. And the study was approved by the ethics committee of the Affiliated Suzhou Hospital of Nanjing Medical University(K-2020-054-K01). Informed consent was obtained from all the individual participants included in the study.

Inclusion criteria: (1) women with PE admitted to our institution for delivery who received IV MgSO_4_ for seizure prophylaxis, (2) aged 18–45 years.

Exclusion criteria: (1) Contraindication to the use of Magnesium (patients with myasthenia gravis or other neuromuscular disorder, severe renal insufficiency, hypermagnesemia, hypocalcemia, hypokalemia, heart block), (2) Specific comorbidity (stillbirth, fetal malformation, diabetes mellitus, hypothyroidism or hyperthyroidism, intrahepatic cholestasis.)

### Administration of MgSO_4_

Patients were administered a 5 g loading dose of magnesium sulfate intravenously over 30–120 min on the first day of administration, followed by a maintenance dose of 10 g over 6–8 h using an infusion pump (Terufusion infusion pump TE-135, Terumo Corporation, Tokyo, Japan). And only a maintenance dose of 10 g was administered on days 2–5. Maternal blood samples were taken at 0, 4, 5, and 12 h after the maintenance dose was administered on day 2 for modeling purposes. The precise timing of blood collection is determined by the prevailing clinical practice. The actual administration rate and the number of administration days are determined by the clinician based on the patient’s condition. Demographic data (maternal age, gestational age, BMI, mode of delivery and adverse reactions (ADR)) and clinical data (creatinine clearance (CCR), albumin, calcium) were obtained at baseline or treatment.

### Assay methods of MgSO_4_

Serum magnesium levels were measured by 2 mL of venous blood sampling, which were determined by VITROS Chemistry Products Mg Slides. The principle is that patient samples are distributed to the reagent layer through the diffusion layer. The magnesium in the sample reacts with the yellow formazan dye in the reagent layer, and the high magnesium affinity of the dye makes magnesium separate from the binding protein. The magnesium-dye complex shifts the maximum spectral absorption, which is directly proportional to the magnesium concentration.

### Population pharmacokinetic model

The PPK analysis was conducted using the non-linear mixed-effect modeling with the Phoenix® NLME software (version 8.3, Certara L.P. United States). The first-order conditional estimation-extended least-squares (FOCE-ELS) method was used to develop the PPK model. The basic model was selected according to the examination of the objective function value (OFV), Akaike information criteria (AIC) and Bayesian information criteria (BIC). According to the selected structural model, the optimal model is selected by the calculation results of additive, proportional and combined random effects models are compared respectively. Meanwhile, the goodness of Fit (GOF) plot was visually examined with reference to previously published research. Then, a stepwise covariate modeling (SCM) method was used to explore candidate covariates on PK parameters, including demographic data (maternal age, diagnosis and BMI), clinical data (creatinine clearance (CCR), albumin, adverse reactions(ADR)) and medications(labetalol, nifedipine, furosemide), where continuous covariates (e.g., BMI) were modeled by using the power function and categorical covariates (e.g. furosemide) were described by a proportional function. The SCM consists of a forward selection step (the criterion is *p* < 0.01 for ΔOFV decreased ≥ 6.63) and a backward elimination step (the criterion is *p* < 0.001 for ΔOFV increased ≥ 10.83.

The final model is checked and evaluated with data and visual. Bootstrap, GOF (including the distribution of residuals) and visual predictive check (VPC) were used to test the model. The GOF was confirmed using the following diagnostic scatter plots: (1) base/final model individual prediction (IPRED) versus observed dependent variable (DV) scatter plot, (2) base/final model population prediction (PRED) versus DV scatter plot, (3) basic/final model corrected weighted residual (CWRES) versus PRED scatter plot, and (4) basic/final model CWRES versus time scatter plot.

To assess the stability of the model, we conducted a nonparametric bootstrap analysis. By generating 1000 replicates using random sampling with replacement, we compare the bootstrap-estimated parameter values with the median and confidence intervals of the final model. Additionally, we fully utilized the VPC option in the Phoenix NLME to perform VPC analysis on the final model. The reliability of our model would be confirmed if the observed DV concentrations fell within the prediction intervals of 97.5th and 2.5th percentile.

### Monte Carlo simulation

Based on the final population model, Monte Carlo simulations with 1,000 replicates were performed for 4 clinically relevant dosing regimens across three levels of renal function (CCR: 140, 175, and 213 ml/min) and three categories of bodily form (BMI: 27, 29, and 33 kg/m^2^). The CCR and BMI were selected using the quartiles of the study’s data The four fixed dosing regimens were a loading dose of 2.5–5 g of MgSO_4_ for 20 min, followed by a maintenance dose of 10 g for 6.7 h or 15 g for 7.7 h. Observe the trend plot of median magnesium concentration over time for different dosing regimens when CCR and BMI are at the median. The percentage of people reaching effective concentration (2-3.5 mmol/L) for each dosing regimen was counted every hour during infusion period (8 h). Dosing regimens were considered appropriate if more than 90% of individuals reaching therapeutic concentrations (2-3.5 mmol/L) of after fourth hours.

## Results

### Study design and demographic analysis

The PPK model for MgSO_4_ used the data of 51 Chinese women, each of whom two to four determinations of plasma concentration were obtained, resulting in a total of 199 plasma concentration values. Table 1 displays the demographic information and biochemical indices of the participants. The pregnant woman had a mean age of 31.86 ± 5.22 years. Among these patients, there were 5(9.80%) patients who attained therapeutic serum magnesium level (2-3.5 mmol/l) within 4–5 h. The baseline magnesium values were 0.76 mmol/L, and 41.20% of the participants experienced ADR related to MgSO_4_, including flush, headache, dizziness, nausea and vomiting. No pregnant women experienced severe adverse reactions such as disappearance of knee tendon reflex or respiratory depression.

**Table 1:** Characteristics of the study population (*n* = 51)

Parameters	Values
Age (years)	31(28,35)
Gestational age (weeks)	32.31 ± 3.92
BMI (kg/m^2^)	29.13(27.16,33.30)
Baseline magnesium concentrations (mmol/L)	0.76(0.71,0.86)
CCR (ml/min)	182.18 ± 67.15
Albumin (g/L)	30.45 ± 4.66
Calcium (g/L)	2.17 ± 0.15
Twins-n (%)	4(7.84)
Labetalol-n (%)	50(98.03)
Nifedipine-n (%)	22(43.14)
Furosemide-n (%)	16(31.37)
**ADR- n (%)**	
Flush	6(28.57)
Headache	5(23.81)
Dizzy	7(33.33)
Nausea	2(9.52)
Vomiting	1(4.76)
Total	21(41.18)
**Mode of delivery- n (%)**	
Cesarean	40(78.43)
Vaginal delivery	4(7.84)
Rivanol Induced Abortion	7(13.73)

BMI Body mass index, ADR Adverse reactions

### Development and evaluation of the MgSO_4_ PPK model

The PPK model of participants administered MgSO_4_ was adequately described by one-compartment structural model, which was used as the base model. And an additive error model was used to evaluate the residual variability, which showed lower AIC、BIC and OFV values. The estimated parameters of the final model are shown in Table 2. Next, the covariate model building identified furosemide, CCR and BMI as the effective covariate for CL, and furosemide as a significant covariate for V. Age, calcium, albumin, labetalol, nifedipine and other clinical variables did not have statistically significant relationships with PK parameters. The estimated parameters and bootstrap replicates 1000 times were presented of the final model are shown in Table 2. The results showed that 95% of the parameters include the original data set parameters, indicating that the model had qualified stability. The CL was estimated as 2.98 L/h and V was 25.07 L. The equations for Vand CL were as follows:

$$\eqalign{{\rm{CL(L/h) = }} & {\rm{2}}{\rm{.98 \times (CCR/51}}{{\rm{)}}^ \wedge }{\rm{0}}{\rm{.39 \times }} \cr & {{\rm{(BMI/51)}}^{ \wedge {\rm{ - }}}}{\rm{0}}{\rm{.54 \times }} \cr & {\rm{(1 - 0}}{\rm{.16 \times furosemide) \times }} \cr & {\rm{exp(\eta CL)}}\,{\rm{V(L) = 25}}{\rm{.07 \times }} \cr & {\rm{(1 - 0}}{\rm{.25 \times furosemide) \times exp(\eta V)}} \cr}$$

**Table 2:** Population PK parameter estimates in the final model and bootstrap

**Parameters(unit)**	Final model results				Bootstrap results			
	Estimate (shrinkage %)	SE(%)	CV (%)	95%CI	Median	SE (%)	CV (%)	95%CI
**Structural model parameters**								
tvV(L)	25.07	0.89	3.54	23.31–26.82	25.04	0.90	3.60	23.21–26.63
tvCL (L/h)	2.98	0.77	25.84	1.29–4.62	2.88	0.96	33.33	1.21–5.12
dVdfurosemide	-0.25	0.044	-17.40	(-0.34)-(-0.16)	-0.25	0.045	-17.84	(-0.33)-(-0.16)
dCLdfurosemide	-0.16	0.031	-19.49	(-0.22)-(-0.096)	-0.15	0.037	-24.36	(-0.23)-(-0.080)
dCLdCCR	0.39	0.038	9.90	0.31–0.46	0.39	0.068	17.34	0.25–0.51
dCLdBMI	-0.54	0.081	-15.15	(-0.70)-(-0.38)	-0.52	0.14	-26.92	(-0.77)-(-0.22)
**Inter-individual variability**								
ω^2^ V (%)	0.023 (13.04)		26.89		0.023		27.53	
ω^2^ CL (%)	0.082 (13.05)		17.38		0.082		19.64	
**Residual variability**								
stdev0	3.65	0.22	6.17	3.20–4.09	3.59	0.24	6.58	3.16–4.09

tv, typical population value; SE, CV, coefficient of variation; d*d*, correction coefficient; 95% CI, 2.5th and 97.5th percentile of the ranked bootstrap parameter estimate; V, volume of distribution; CL, clearance; ω inter-individual variation; stdev0, standard deviation

**Fig. 1:**
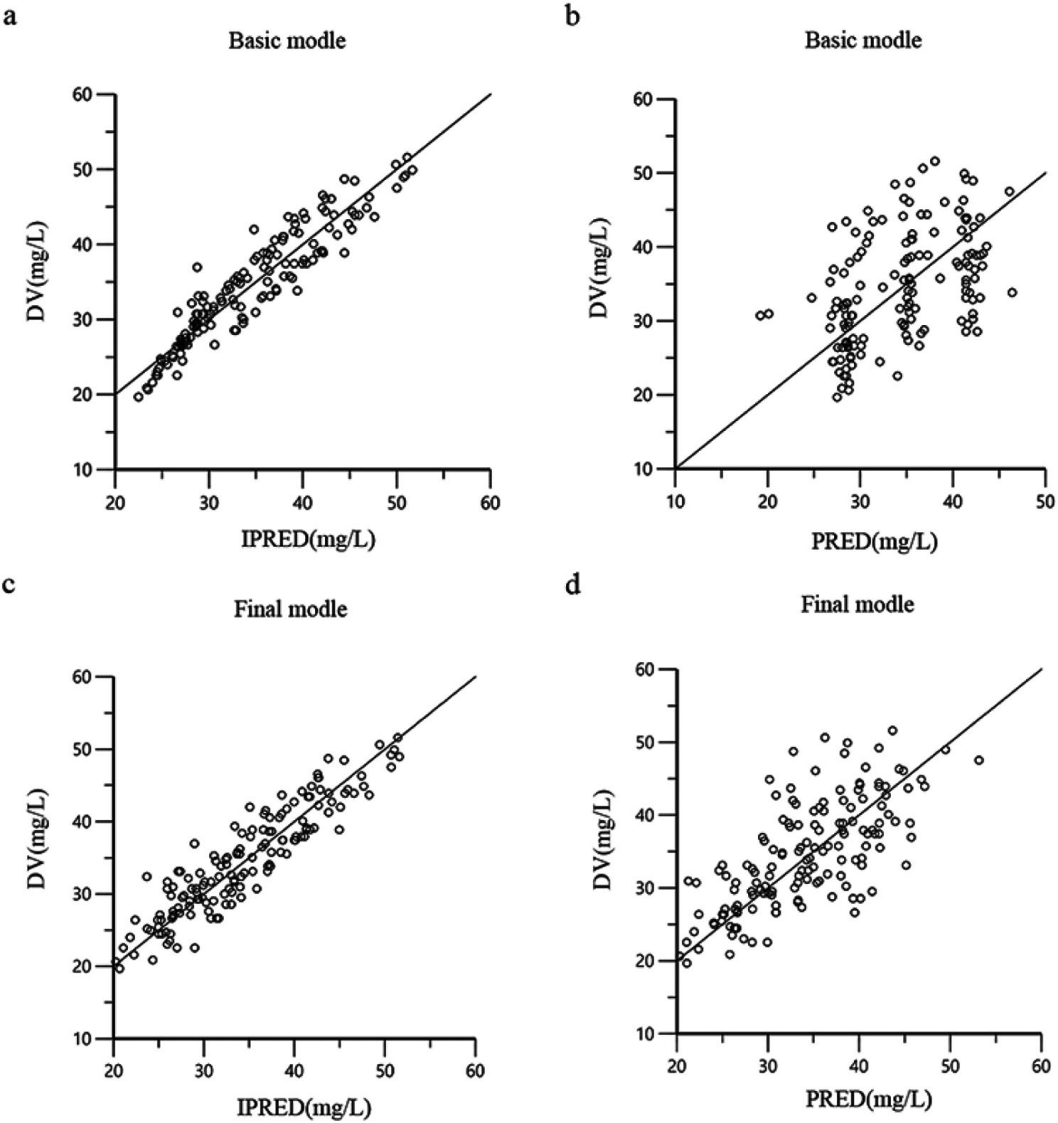
**a** Observed MgSO4 concentrations vs. individual-predicted MgSO4 concentrations for basic model. **b** Observed MgSO4 concentrations vs. population-predicted MgSO4 concentrations for basic model. **c** Observed MgSO4 concentrations vs. individual-predicted MgSO4 concentrations for basic model and final model. **d** Observed MgSO4 concentrations vs. population-predicted MgSO4 concentrations for final model

**Fig. 2:**
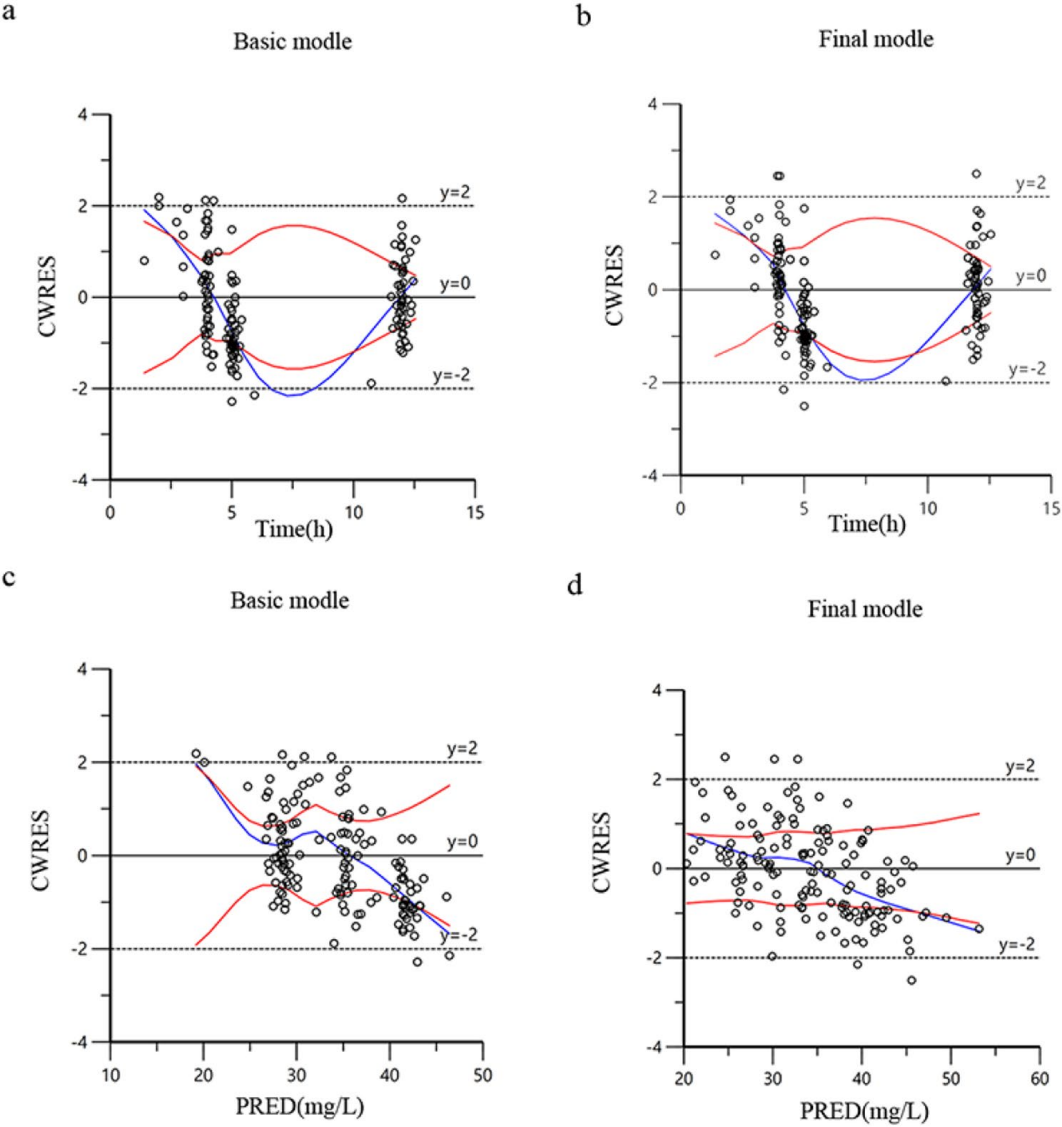
**a** Conditional weighted residuals vs. time (CWRES-*Vs.* IVAR) for basic model. **b** Conditional weighted residuals vs. time (CWRES-*Vs.* IVAR) for final model, **c** Conditional weighted residuals vs. population predicted concentrations (CWRES *Vs*. PRED) for basic model. **d** Conditional weighted residuals vs. population predicted concentrations (CWRES *Vs.* PRED) for final model

**Fig. 3:**
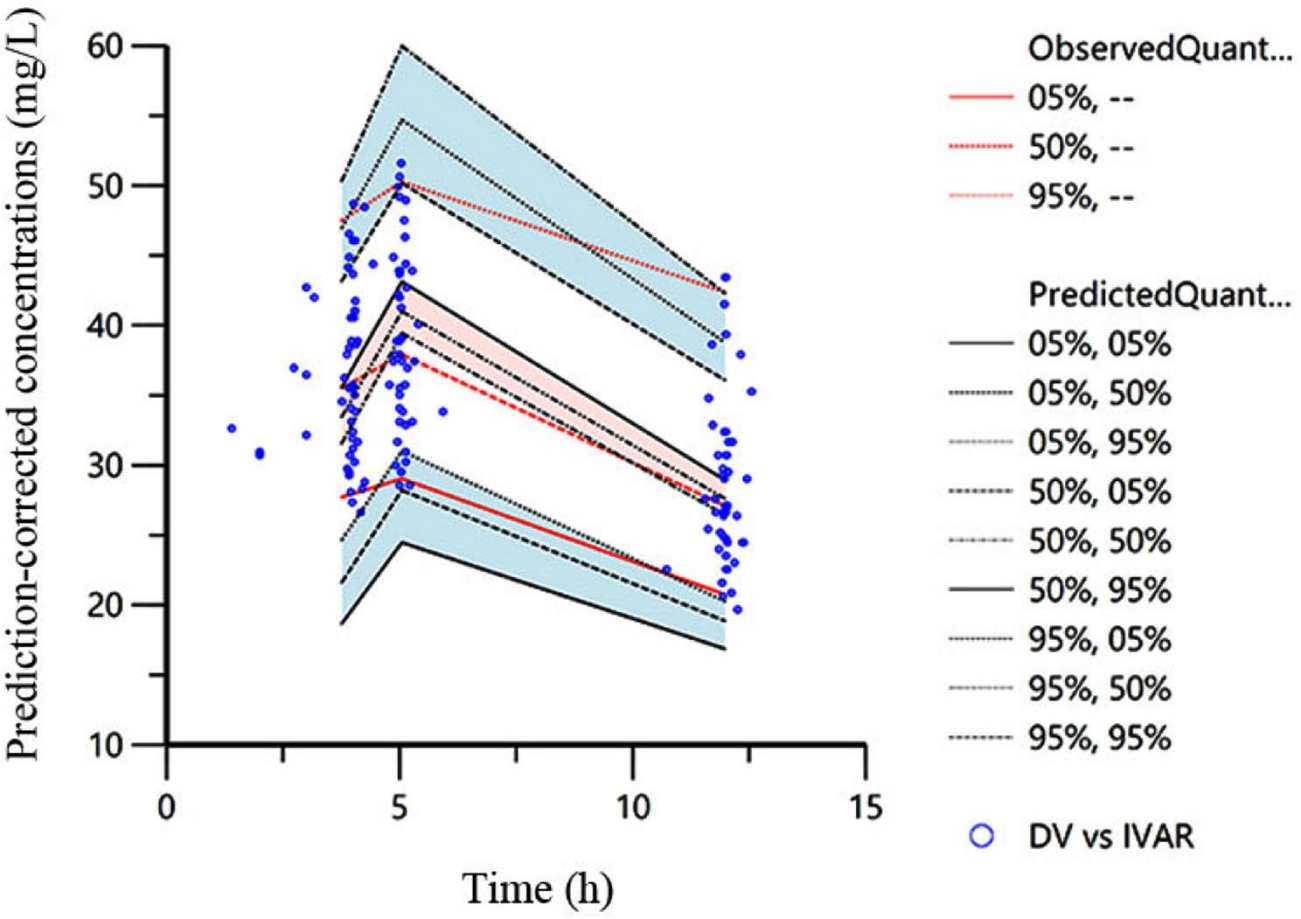
Predicted versus observed concentrations of MgSO_4_ (VPC). The dotted lines represent the 5th, 50th, and 95th percentiles of the simulated data

The actual observed plasma concentrations correlated well with the population and individual plasma concentrations predicted by the final model (Fig. 1). Conditional weighted residuals (CWRES) had a better fit in the final model compared to the performance of the base model, but it still had an obvious trend, suggesting that there may be important factors that are not found to affect the accuracy of the model, and most value of CWRES was between ± 2 with few outliers (Fig. 2). In the VPC, the observed data and the simulation have similar distribution characteristics near the 95th and 5th percentile, but the distribution difference was slightly greater near the median. In general, the model can accurately describe the original data features (Fig. 3).

### Monte Carlo simulations

**Fig. 4:**
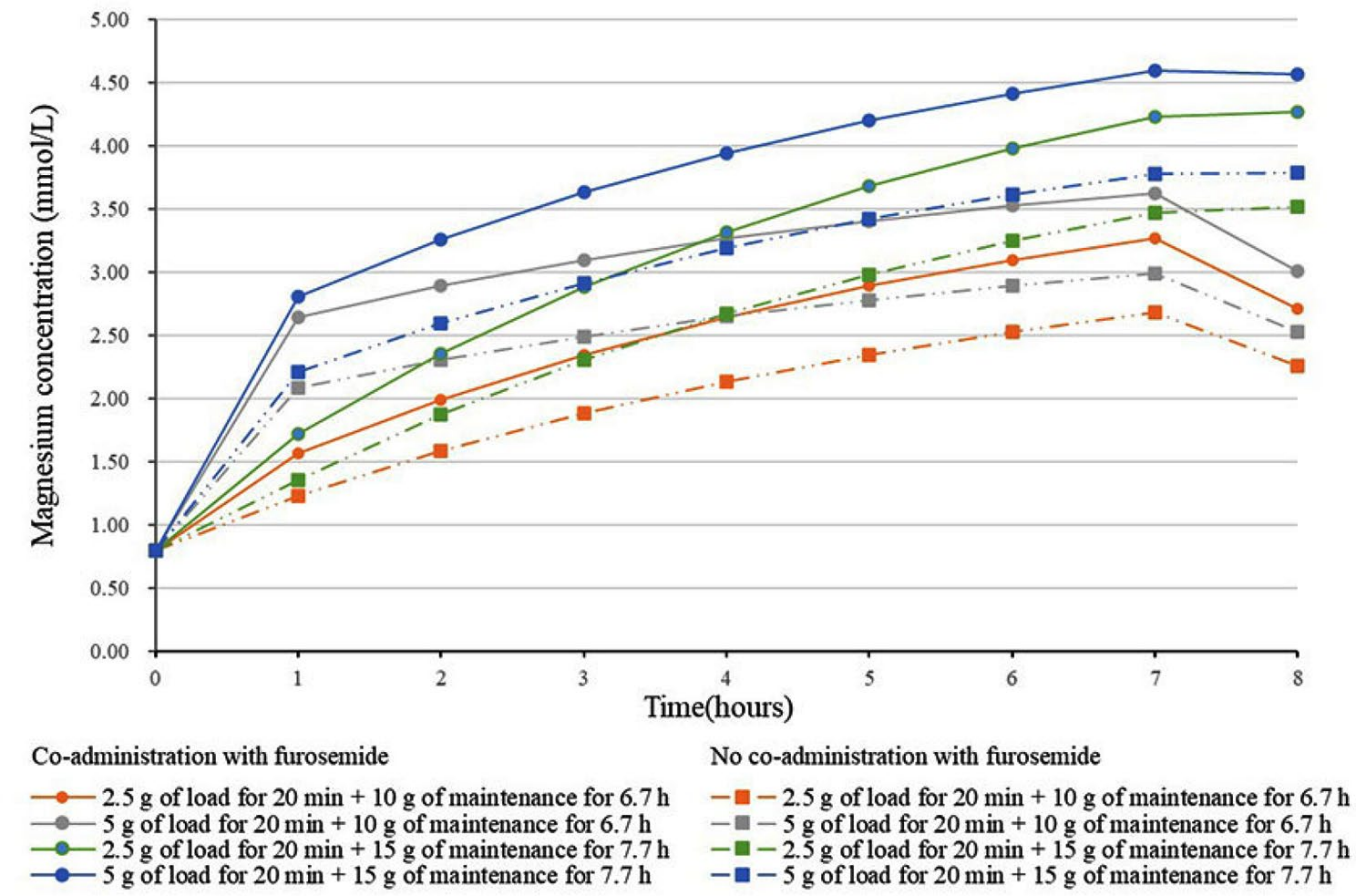
Median magnesium concentration over time for each time period for different dosing regimens when CCR and BMI of simulated patients are median

The hourly calculation of the percentage of individuals falling within the therapeutic concentration range was conducted for various MgSO_4_ treatment regimens. Figure 4 presents the median simulated magnesium concentration over time for different dosing regimens when CCR and BMI are taken at the median (CCR:175 ml/min, BMI:29 kg/m2). A loading dose of 5 g ensures rapid attainment of therapeutic concentrations within one hour. In the same dosing regimen, patients received furosemide had higher blood magnesium concentrations than those who did not receive furosemide.

**Fig. 5:**
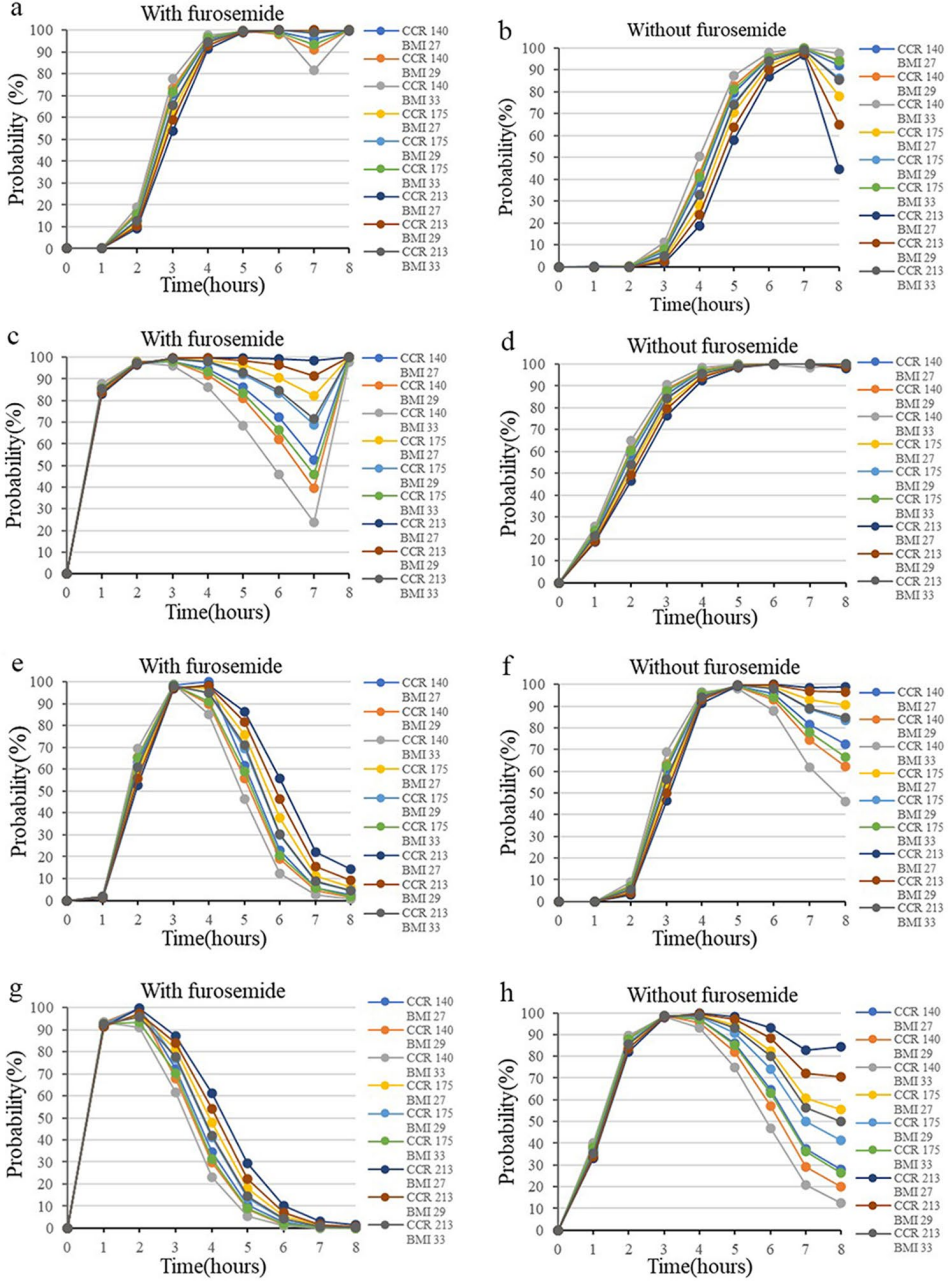
Probability of target achievement (PTA) for different dosage regimens of MgSO4 for targets with 2.0-3.5mmol/L. (**a-b**) loading dose of 2.5 g for 20 min, followed by a maintenance dose of 10 g for 6.7 h. (**c-d**) loading dose of 5 g for 20 min, followed by a maintenance dose of 10 g for 6.7 h. (**e-f**) loading dose of 2.5 g for 20 min, followed by a maintenance dose of 15 g for 7.7 h. (**g-h**) loading dose of 5 g for 20 min, followed by a maintenance dose of 15 g for 7.7 h

As Fig. 5 presented, for the group receiving furosemide (a, c, e, g), a loading dose of 2.5 g followed by a maintenance dose of 10 g was appropriate for patients in various CCR and BMI categories, except for those with CCR < 140 ml/min and BMI > 33 kg/m^2^ (a). In particular, in individuals with CCR > 213 ml/min and BMI between 27 and 29 kg/m^2^, the 5 g loading dose and 10 g maintenance dose regimen allowed patients to rapidly reach therapeutic concentrations within two hours compared to the 2.5 g loading dose regimen(c). For the group not receiving furosemide (b, d, f, h), a loading dose of 5 g followed by a maintenance dose of 10 g was deemed appropriate(d). The dosing regimen of a 2.5–5 g loading dose and a 15 g maintenance dose was not adequate for all populations due to significantly increased toxicity(e-h), except for those with CCR > 213 ml/min and BMI between 27 and 29 kg/m^2^(f).

## Discussion

This study established a PPK model to obtain the relevant pharmacokinetic parameters and performed Monte Carlo simulations and dose optimization. The results showed that fewer patients reached the effective therapeutic concentrations. Finally, a one-compartment PPK model with three covariates was developed and evaluated. The model estimate of the population CL was 2.98 L/h; for V, it was 25.07 L. The V was adjusted for furosemide and Magnesium clearance was further adjusted for CCR, BMI and furosemide. Optimizing the dose depended on whether furosemide is combined or not. This study is the first to investigate the pharmacokinetics of MgSO_4_ in a Chinese population with PE and the first global study to conduct PPK modeling during maintenance dose administration of MgSO_4_. Additionally, it is the first study to report that the co-administration of furosemide has a significant impact on the PPK model of MgSO_4_.

A possible explanation for the dramatic differences in the pharmacokinetics of MgSO_4_ between countries could be the presence of comorbidities or underlying medical conditions in the patient population, which may affect the distribution and clearance of MgSO_4_. Furthermore, individual variations in genetic factors, such as different races, can also influence the MgSO_4_ clearance. The variability described in Table 2 indicates that empirical dosing, without considering individual serum magnesium concentrations, is unlikely to be appropriate for most patients. A pharmacokinetic model appropriate for the Chinese population is necessary to guide the rational administration of magnesium sulfate. In Chinese clinical practice, a loading dose of MgSO_4_ is usually only administered to the gravida who begin to receive treatment of MgSO_4_ on the first day [9]. The study found that the blood magnesium concentration before the second day of administration was 0.76 mmol/L, which is consistent with previous reports on the baseline blood magnesium concentration in patients with PE ranging from 0.58 to 1.0 mmol/L [10]. It can be inferred that the MgSO_4_ administered on the first day of treatment was mostly excreted. Therefore, the probability of achieving therapeutic concentration in this study was lower at 9.80% compared to studies where loading doses were administered [11, 12]. Magnesium disposition is described adequately by a one-compartment model in our study, which is consistent with 5 reports in the literature [7, 13–16]. Recent studies on the PPK of MgSO4 have reported that the volume of magnesium distribution ranged between 13.3 and 65.1 L and the value of CL in the population ranged between 1.38 and 5.00 L [11, 13–18]. Our study found the CL value of 2.98 CL/h, which is lower than the values typically described in literature. However, this difference may be attributed to the lower levels of CCR in the patients we evaluated. The value of V is accordance with that found in the study published by Brookfield et al [11].

In our study, CCR, BMI, and the use of furosemide have been shown to be important parameters to be considered in individualized therapy. During the normal course of pregnancy in women, the glomerular filtration rate and renal plasma flow increase by 40–65% and 50–85%, respectively. Consequently, the CCR in our study population was higher than that observed in normal women [19]. Magnesium ions are not metabolized, making MgSO_4_ a drug that is entirely excreted by the kidneys. The CCR is one of the indicators of renal function, and when it is low, it suggests impaired renal function. A low CCR suggests impaired renal function, which can affect the rate of renal excretion of drugs, leading to their accumulation in the body. This may explain the higher blood magnesium concentrations in women with preeclampsia who have a low CCR, and may also be a factor in modulating CL values.

In clinical practice, furosemide is used to correct patients with PE with symptoms of volume overload (including anasarca, pulmonary edema, cerebral edema), elevated BNP, oliguria and severe hypoproteinemia [9, 20]. Interestingly, the effect of furosemide on blood magnesium concentration in this study appears to be related not only to the state of progression of the disease, but also to its pharmaco-mechanism of increasing electrolyte and water excretion. Diuretics are the cornerstone of therapy for volume overload [21]. Furosemide is typically used for clinical indications related to renal impairment and water retention caused by disease progression. which may result in the retention of last administered magnesium ions due to the inability to excrete them, leading to higher blood magnesium concentrations in patients receiving furosemide than in those not receiving furosemide. Loop diuretics reduce hypervolemia and severe fluid overload [22], consequently, of the V. In a doubleblind placebo controlled study, furosemide administration can increase the excretion of magnesium ions after a single oral dose, it may not be sufficient to bring about a decrease in blood magnesium due to its delayed effect and overall compensatory response of 24 h [23, 24].

The final parameter that affects CL is BMI. BMI is an international indicator used to measure the degree of fat and thinness of the human body and determine whether it is healthy, and is calculated using a formula that includes weight and height. The results of our study indicated that body weight was not a significant covariate for pharmacokinetic parameters. However, there was a positive correlation between BMI and drug concentrations, which is inconsistent with other studies showing that body weight is associated with lower drug concentrations [11, 18, 25]. Differences in the subtypes of PE and abnormal weight gain associated with preeclampsia due to disease progression may account for this diametrically opposed phenomenon [26, 27]. In the subtype of PE associated with vasculature and high cardiac out, obesity predisposes to enhanced water retention, which may further lead to venous congestion, responsible for ascites, eclampsia, or placental abruption [28]. This could explain the elevated BMI associated with water retention and lead to accumulation of blood magnesium concentration.

Supratherapeutic level of magnesium sulphate can lead to toxic reactions. Monitoring the patellar reflexes and avoiding concentrations beyond the therapeutic range have been the traditional clinical practice for addressing this issue. However, this traditional approach fails to address the issue of the achievement of a specific therapeutic concentration. Encouragingly, PPK and Monte Carlo simulation provide effective solutions to this difficult problem. Only 9.80% of subjects in this study achieved the therapeutic window at 4–5 h, indicating that a sole maintenance dose regimen would not attain the desired effect. Adjusting the loading dose and shortening its administration time could enable the target population to reach the desired blood magnesium concentration, as assessed and analyzed through Monte Carlo simulations. The grouping of furosemide, a categorical covariate, can effectively co-ordinate the different types of CCR and BMI patients to determine the appropriate dosing regimen. This may be due to the fact that furosemide use may be accompanied by abnormally high BMI and decreased renal function. The optimal dosing regimen can be identified through this Monte Carlo simulation in all patients, with the exception of those taking furosemide with CCR < 140 mL/min and BMI > 33 kg/m². It can be concluded that patients taking furosemide can use the dosing regimen of a loading dose of 2.5 g in combination with a maintenance dose of 10 g as an initial treatment regimen for optimal efficacy, with the exception of patients with CCR of less than 140 ml/min and BMI of greater than 33 kg/m². And a 5 g loading dose and a 10 g maintenance dose may be used as the initial treatment regimen for those not taking furosemide. After dose optimization based on the established model, the actual clinical efficacy of the recommended dosing regimen is assessed in clinical practice based on the model that has been created.

Our results confirm the effects of BMI, furosemide and CCR on CL and V. The equation includes both continuous and categorical covariates. We recommend the model for women with furosemide because of their Supratherapeutic risk, as well as for women with extreme BMI and renal clearance. This study validates some of the results of other studies and adds new covariate data. However, our study has some limitations. Blood samples are not taken after 4 to 5 half-lives due to limitations in sample collection. In addition, the established pharmacokinetic model of MgSO_4_ in Chinese patients with PE was only internally evaluated and did not undergo further external evaluation, which requires assessment and evaluated in future studies.

## Conclusions

The study characterized the PPK of MgSO_4_ when administered to patients with PE. It identified furosemide, the CCR, and BMI as significant covariates that explain interindividual pharmacokinetic variability. It is important to note that furosemide affects both the V and CL, which has not been observed in previous studies. Initial regimen selection can be made based on the use of furosemide or not. But the model evaluation diagram indicates that there are unknown factors affecting the accuracy of the model, which also suggests that further studies should be expanded and external evaluation should be carried out to find the sources of affecting the stability of the model.

## Data Availability

The data of this study is available from the corresponding authors on reasonable request.
